# Effects of Oral Sodium Bicarbonate Supplementation on Protein Metabolism and Inflammation in Iraqi Hemodialysis Patients: An Open-Label Randomized Controlled Trial

**DOI:** 10.1155/2023/6657188

**Published:** 2023-07-28

**Authors:** Zina A. Rasheed, Ban A. AL-Hashemi, Ala A. Ali

**Affiliations:** ^1^Nephrology and Renal Transplantation Centre, Baghdad Medical City, Baghdad, Iraq; ^2^Baghdad Al-Russafa Health Directorate, Baghdad, Iraq

## Abstract

**Background:**

The effect of correcting metabolic acidosis on protein metabolism in hemodialysis patients is controversial.

**Objectives:**

To study the effects of oral sodium bicarbonate on protein metabolism and markers of inflammation in acidotic hemodialysis patients. *Patients and Methods*. An open-label randomized controlled trial was conducted at a single center. Sixty-six clinically stable adult hemodialysis patients were recruited with an average predialysis serum bicarbonate level of <22 mmol/l and a dialysate bicarbonate concentration of 35 mmol/l. Forty-nine participants have completed the study. Oral sodium bicarbonate tablets of 500 mg were given daily in the intervention group (*n* = 25) for 12 weeks versus the standard of care in the control group (*n* = 24). Outcomes compared intervention versus nonintervention in both groups at equivalent time points (0 and 3 months). The clinical data, anthropometry, dialysis adequacy, albumin, normalized protein catabolism rate, blood gas analysis, and bicarbonate were recorded at 0 and 3 months. In addition, muscle mass and handgrip strength were measured. Finally, IL-6 as a marker of inflammation was measured at randomization and three months.

**Results:**

Serum bicarbonate and pH increased significantly from 17.57 ± 3.34 mmol/L to 20.69 ± 2.54 mmol/L and from 7.26 ± 0.06 to 7.34 ± 0.04, respectively (*p* < 0.0001). Serum albumin was significantly higher in the intervention group at three months than in the control group, 4.11 ± 0.45 vs. 3.79 ± 0.47 (*p* value 0.011). Serum potassium significantly decreased in the intervention group at three months compared to the control group, 5.00 ± 0.43 mEq/l vs. 5.33 ± 0.63 mEq/l (*p* value 0.03). Muscle strength expressed as handgrip has improved significantly in the intervention group at three months compared to the control group, 45.01 ± 19.19 vs. 33.93 ± 15.06 (*p* value 0.03). The IL-6 values were less in the intervention group at 3 months with a *p* value of 0.01. The interdialytic weight of the intervention group at three months was 2.42 ± 0.64 compared to the 2.20 ± 1.14 control group, but this did not reach statistical significance (*p* value of 0.4). The composite of (albumin + nPCR) at three months was achieved in 59.18% of the intervention group compared to 14.28% with a *p* value of 0.01.

**Conclusions:**

Correcting metabolic acidosis in hemodialysis patients improved serum albumin and nPCR without hypokalemia or significant interdialytic weight gain. This was particularly evident in patients with minimal inflammation with low IL-6 values.

## 1. Introduction

End-stage kidney disease (ESKD) is a worldwide health problem with 11–13% global prevalence, causing a significant health care and economic burden. Renal transplantation is the best form of renal replacement therapy as it provides a survival benefit over remaining on dialysis [1].

Historically, the uremic milieu was always regarded as a state of acidosis, and it is currently termed the metabolic acidosis (MA) of chronic kidney disease (CKD). MA is usually evident but unaccompanied by signs or symptoms of uremia. Regardless of the term, it is defined as persistently low bicarbonate levels of less than 22 mEq/L [2]. It is usually evident when the glomerular filtration rate (GFR) is lower than 30 mL/min/1.73 m^2^. Below this level of filtration, the body may tolerate acute acidemia but cannot completely correct the base deficit on chronic bases [3].

The prevalence of MA is approximately 15–19% in CKD stage 3–5 patients, which tends to increase with the progressive loss of GFR. Furthermore, studies reveal the importance of MA in the etiopathogenesis of CKD patients' increased morbidity and mortality [4, 5].

Metabolic acidosis of CKD/ESKD leads has a detrimental effect on multiple metabolic pathways. These include increased parathyroid hormone synthesis, bone resorption, altered glucose metabolism, and increased protein catabolism. The latter leads to MIA (malnutrition-inflammation-atherosclerosis) syndrome and sarcopenia [6, 7].

Acid directly stimulates hepatic glutamine production and accelerates muscle protein degradation, releasing amino acids to synthesize glutamine in the liver. Moreover, acidemia increases the oxidation of branched-chain amino acids (valine, leucine, and isoleucine) in muscle to provide much of the nitrogen used in the hepatic synthesis of glutamine. It activates the rate-limiting enzyme for the irreversible decarboxylation of BCAA and branched-chain ketoacid dehydrogenase (BCKAD) in muscle, and this response accounts for BCAA degradation and decreased albumin synthesis [7].

Animal studies proved the proteolytic effect of MA [8]. It is both catabolic and antianabolic, and it acts synergistically with other catabolic factors, such as inflammatory cytokines and insulin resistance, inducing protein catabolism and increasing the risk of malnutrition [9].

Metabolic acidosis also contributes to the inflammatory milieu of uremia with upregulation of inflammatory cytokines like IL-6 and consequent proteolysis and loss of muscle mass [10]. Thus, correction of MA may, at least, partially alleviate the deleterious effect of inflammation on muscle protein synthesis.

In CKD patients, bicarbonate supplementation slows the progression of kidney disease to ESKD. It improves nutritional status [11]. Correction of MA in HD patients effectively improves nutritional status. This can be achieved by higher dialysate bicarbonate or an oral bicarbonate supplement [12]. In chronic ambulatory peritoneal dialysis (CAPD) patients, correction of acidosis decreased the whole body degradation [13]. Thus, bicarbonate supplements can be given to maintain serum bicarbonate of 24_26 mEq/L (KDOQI Clinical Practice Guideline FOR Nutrition in CKD 2020 update) throughout the interdialytic interval to compact the harmful effect of uncorrected acidosis [14].

In Iraq, there are about 7000 prevalent HD patients. In a recent multicenter study, 49.8% of Iraqi adult HD patients were malnourished [15].

This study investigates the effects of supplementing oral sodium bicarbonate in acidotic hemodialysis patients on protein metabolism and markers of inflammation.

## 2. Patients and Methods

An open-label randomized controlled trial was conducted in a single governmental hemodialysis unit from August 1, 2021, to December 31, 2021.

Patients: adult ESRD patients on regular HD for >3 months. Eligibility criteria include three times hemodialysis a week, documented serum bicarbonate level of <22 mmol/l, with a dialysate bicarbonate concentration of 35 mmol/l, no residual renal function (24 hours urine output <200 ml), arteriovenous fistulae as dialysis access and the patients should be in a steady clinical state and willing to provide written informed consent.

The exclusion criteria were as follow:

1. Acute illness or infection in the last three months, including coronavirus (COVID-19)
2. Patients with an anticipated life expectancy of 6 months (e.g., due to metastatic malignancy or terminal disease)
3. Advanced senility and impaired cognition
4. Clinically evident cachexia and sarcopenia
5. Ongoing enteral or parenteral nutrition
6. Patients with uncontrolled blood pressure (>160/90)
7. Patients who primarily have predialysis potassium levels of less than 4 mmols/L
8. Use of steroids or immunosuppressive agents
9. If the patient is already using oral sodium bicarbonate therapy.

Sixty-six patients fulfilled the eligibility criteria. Seven patients were transferred to other satellite dialysis units that could not be followed, whereas the other nine refused to consent.

### 2.1. Hemodialysis Protocol

Patients receive three weekly dialysis sessions, 4 hours each, using a Baxter Polyflux 21 L (Low flux) dialyzed. The dialysis blood flow (Qb) ranges between 230 and 300 ml/min, while the dialysate flow (Qd) ranges between 550 and 750 ml/min. Still, HDF is not widely applicable in Iraqi dialysis practice.

### 2.2. Randomization

The study participants were randomly allocated in 1 : 1 ratio to intervention (*N* = 25) and control (*N* = 25) groups by a nonresearch team health personnel sealed envelopes. Enrolled patients were stratified by nPCR.

### 2.3. Intervention

Patients in the intervention group were assigned to receive oral sodium bicarbonate tablets 500 mg fixed dose daily. The unit-specialized pharmacist assessed adherence to the prescribed dose of sodium bicarbonate and reported the medication records, the home use of the oral capsules, and drug-related side effects.

### 2.4. Data collection

The medical history and sociodemographic characteristics were recorded by direct interviewing the study participants. The dialysis and laboratory data were collected from the patient's charts and records. Blood pressure values were taken as the mean of predialysis measurements of the last three dialysis sessions. In addition, anthropometric measurements were recorded.

The baseline biochemistry data included serum bicarbonate, arterial pH, renal function, serum sodium, and serum potassium. Serum albumin was measured by the green bromocresol method. Blood samples were taken after the longest interdialytic interval for pre- and postdialysis urea, and the Kt/V and normalized protein catabolic rate (nPCR) were calculated. In addition, IL-6 levels were measured at baseline and three months using a Roche Cobas e411 (Roche Diagnostics GmbH, Mannheim, Germany, 2020).

Values of acid-base parameters were derived from the blood gas analysis performed as a specific study procedure using IRMA (Blood Analysis System/2018/Germany). Samples of 2 mL were collected in heparinized syringes before a midweek dialysis session. Samples were collected from the indwelling needle puncturing the arteriovenous fistula's arterial limb. After collection, the syringe was immediately transported to the laboratory.

## 3. Operational Definitions

### 3.1. Anthropometric Data

- Height measurement, based on recently documented measures from the patient's record
- Weight measurement, based on the documented last postdialysis weight measurements
- Body mass index (BMI) was calculated by dividing the dry weight over the squared height in meters
- Interdialytic weight gain was calculated as the patient's weight at the beginning of each HD session (predialysis weight) minus the weight after (postdialysis weight) the previous HD session.

### 3.2. Dialysis Adequacy [16]

Dialysis adequacy was estimated by calculating midweek, single pool Kt/V according to

(1) $$\begin{array}{c} Daugirda\;s\;equation:\frac{spKt}{V}=-\ln\left(R-0.008\;\times \;t\right)+\left(4-3.5\;\times \;R\right)\;\times \;\frac{UF}{W}\text{,} \end{array}$$

where *R*: predialysis urea/postdialysis urea, *t*: dialysis time in hours, −ln: negative natural logarithm, UF: ultrafiltration volume, *W*: the body weight after the HD session, and a Kt/V of 1.2 was considered the minimum clinically acceptable value in our dialysis unit.

### 3.3. The Normalized Protein Catabolic Rate (nPCR)

The protein catabolic rate (PCR) reflects the amount of protein catabolized more than the amount synthesized daily, determined in stable chronic hemodialysis patients to evaluate dietary protein intake [17].

nPCR was calculated using two equations, and the average was taken in our study [18].

- nPCR, in g/kg per day = 0.22 + (0.036 × ID rise in BUN × 24)/ID interval (hours) where the interdialytic (ID) rise in BUN (predialysis BUN minus the one-to-two-minute postdialysis BUN from the preceding dialysis) is expressed in mg/dL and ID interval: intradialytic interval (hours) [19, 20].
- nPCR = (0.0136 × [Kt/V × ([predialysis BUN + postdialysis BUN] ÷ 2)]) + 0.251.

Daily protein intake of 1.1–1.2 g/kg/day was recommended in HD patients in our study, so less than 1 g/kg/day was regarded as poor nutritional status.

### 3.4. Muscle Function and Strength

Hand Dynamometer Grip Strength Measurement (CAMRY DIGITAL HAND DYNAMOMETER, EH101, USA): measured before dialysis; by squeezing the hand dynamometer with maximum effort for at least 5 seconds and repeated three times by a nonfistulous arm. After testing, the maximum grip value is considered, and a grip value status bar shows the status of “weak,” “normal,” or “strong” according to age and gender preset for each test.

### 3.5. Skinfold Thickness

Triceps skinfold (TSF) thickness (XTDGN Skinfold Body Fat Caliper, China): the halfway distance between the acromion and the olecranon (posterior surface) was marked. After holding a fold of skin, the TSF measurement was taken using a Lange skinfold caliper, with the caliper's jaws at the level of the marked skin.

The handgrip strength and triceps skinfold thickness were measured by an independent examiner expert in using these tools blinded to treatment status.

## 4. Outcome

The primary endpoint is to assess the efficacy of sodium bicarbonate therapy in improving nutritional parameters {nPCR with a target of 1.2 (g/kg per day) and serum albumin with a target of 4 (gm/dl)} and to ameliorate inflammation by reducing IL-6. The secondary endpoint is to assess intervention safety regarding volume retention and hypokalemia.

## 5. Administrative and Ethical Approval

The ethical and scientific committee of the Arab Board for Health Specialties–Clinical Nutrition fellowship program and the Ministry of Health Ethics Committee approved the study protocol in July 2021 (Number: 2021032/Baghdad). The study's objectives and the confidentiality of the data were explained to the participants. According to the National Research Ethics Code, all participants provided written informed consent.

## 6. Statistical Analysis

Data were entered and analyzed using IBM SPSS Statistics for Windows (version 24.0, IBM Corp., Armonk, NY). Descriptive statistics are presented as percentages, frequencies, means, and standard deviations according to the variable type. The differences between and within groups were compared using the *t*-test and chi-square tests. For variables with skewed values, a Mann–Whitney test was used. Because of the small sample size and the possible imbalances between some outcomes, we used repeated measure ANOVA to account for differences in the baseline characteristics. A bivariate Pearson correlation was used to assess the correlation between two numerical variables. Statistical significance was set at *p* < 0.05.

## 7. Results

**Figure 1:**
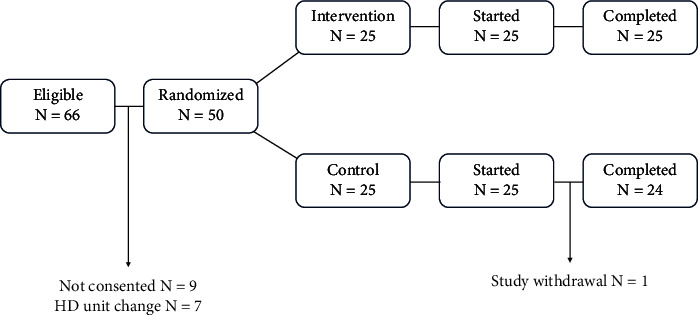
The flow of the study participants.

Of the sixty-six eligible patients, fifty were recruited (age range 20–80 years, 55.1% female). However, after randomization, one participant from the control group withdrew according to his will (Figure 1).

Table 1 represents the baseline characteristics of the study groups at time 0.

**Figure 2:**
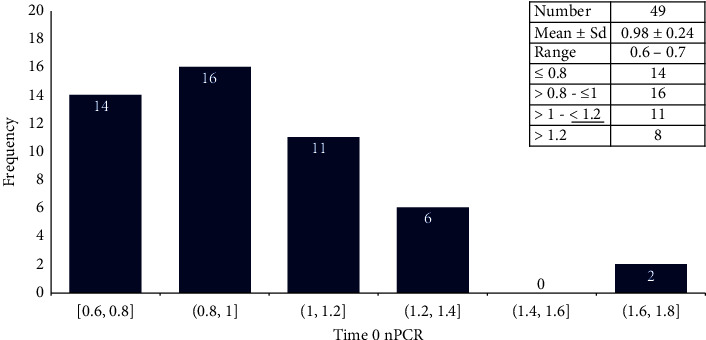
The distribution frequency of the study participants by (time 0 nPCR).

The participants were in good nutritional status with normal BMI and serum albumin. The mean nPCR for both groups was 0.98 + 0.24 g/kg/day, below the recommended target by most guidelines. Twenty-nine participants (29/49, 59.18%) were with values <1 g/kg/day (Figure 2).

The arterial pH significantly increased at three months in the intervention group compared to the control group (*p* value 0.001). This also applies to serum bicarbonate at three months compared to baseline and the control group (*p* value <0.001 and 0.05, respectively).

Table 2 represents the intervention and control groups' clinical, laboratory, and nutritional data at three months of oral sodium bicarbonate therapy. Considering any imbalances between the groups in some of the outcomes, repeated measures ANOVA was used, and the results are represented in Table 3.

## 8. Primary Outcome

There was a statistically significant difference in the nPCR in the intervention group at three months (*p* value 0.03) and, in comparison, to the control group at three months (*p* value 0.033). In addition, the serum albumin was significantly higher at three months in the intervention group than in the control group (*p* value 0.011).

Supplementing acidotic hemodialysis patients with oral sodium bicarbonate improved protein metabolism by increasing serum albumin and nPCR. Serum albumin increased in the intervention group with a relative risk of 3.75, *p* value of 0.006; the nPCR increased in the intervention group with a relative risk of 4.48, *p* value of 0.008 (Table 4).

**Figure 3:**
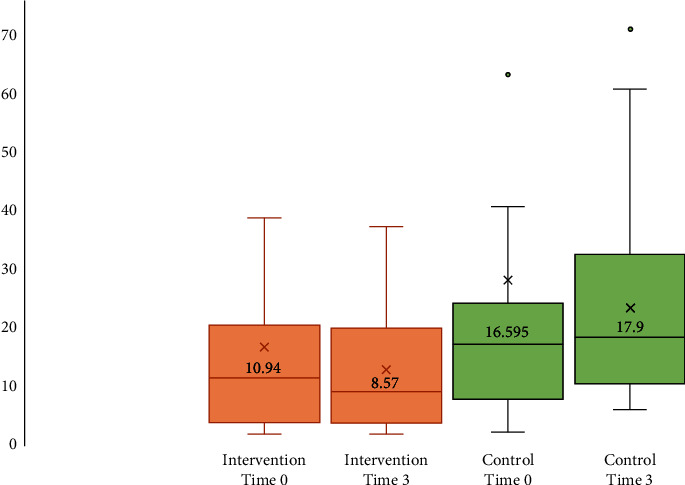
The mean IL-6 values at 0 and 3 months for the intervention and control groups.

The IL-6 values were highly skewed; thus, a Mann–Whitney test was used to assess the difference between time 0 and 3 months. The exact 2-sided value was 0.009, and the *p* value was 0.01 (Figure 3).

Bivariate Pearson's correlation analysis revealed no correlation between serum albumin and IL-6 with blood PH and bicarbonate in the intervention group at three months.

Muscle strength expressed as handgrip has improved significantly in the intervention group at three months (*p* value 0.001) compared to the control group (*p* value 0.03).

## 9. Secondary and Other Outcomes

At three months, the intervention group showed a small but significant increment in serum sodium from time 0 (*p* value 0.034). Still, there was no statistically significant difference from the control group at three months (*p* value 0.28).

The intervention group showed a small increment in body weight at three months (*p* value 0.03). Still, it was not statistically significant compared to the control group at three months (*p* value 0.29). Furthermore, it was not reflected in a substantial Interdialytic weight gain (*p* value 0.405). In addition, both study groups had no statistically significant differences in systolic and diastolic blood pressure at three months.

Serum potassium significantly decreased in the intervention group at three months (*p* value <0.001) compared to the control group (*p* value 0.03).

## 10. Discussion

In this study, a fixed oral dose of sodium bicarbonate, 500 mg/day, has significantly corrected acidosis and improved nPCR with a possible reduction of an inflammatory marker.

Moderate to severe MA in HD patients exert a detrimental effect on serum albumin concentration, partially independent of protein intake as evaluated by nPCR, taking into consideration other nutritional parameters of concomitant inflammatory status as reported by Movilli et al. [21]. Correction of acidosis is one of the primary purposes of renal replacement treatment. However, interventional studies of correcting MA in HD patients generated conflicting results regarding the ability to improve serum albumin concentration, protein degradation, or nutritional status [11, 22].

The mean nPCR for the study groups was 0.98 + 0.24 g/kg/day, and it may reflect better dialysis management than previous Iraqi data with nPCR of 0.87 ± 0.24 g/kg/day [23]. Still, the nPCR of Iraqi maintenance HD patients is below the recommended targets and could reflect significant protein restriction in Iraqi HD practice. A previous report by William et al. demonstrated that in CKD/ESKD patients, the metabolic adaptation to low-protein diets is impaired. Such patients should not be prescribed a restricted protein diet without correcting acidosis. If a low-protein diet is prescribed in these circumstances, it will likely be less efficient in achieving its primary objectives. It could also simultaneously expose the patient to the risk of the accelerated loss of lean body mass [24, 25].

At a steady state, nPCR is assumed to be approximately equal to dietary protein intake. It is used as an objective tool to quantify protein intake and patients' compliance with the dietary prescription in HD patients. It may provide an index of protein catabolism. It does not differentiate between proteins derived from dietary sources or the catabolism of endogenous proteins. In the current study, correction of acidosis has increased nPCR in concordance with Bastani et al. [26, 27].

The low serum albumin concentration in HD patients is an early and sensitive marker of protein malnutrition and mortality. It is also a negative acute-phase reactant that decreases in response to inflammation [28, 29]. The correction of MA in the intervention group was associated with a significant increase in serum albumin concentration. It is either due to acidosis correction or decreased inflammation, as indicated by lower IL-6 in this group. Brady et al. concluded that correcting acidosis does not affect serum albumin levels. These findings suggest that the correction of MA in patients with only minimal or absent chronic inflammation may positively affect serum albumin concentrations. In contrast, in those with more pronounced chronic inflammation, this condition's inhibitory effect on albumin synthesis prevents serum albumin concentrations from increasing, as Movilli et al. [22, 30]. There was no linear correlation between serum albumin and bicarbonate or IL-6. Larger cohorts and longer follow up should provide better data.

Although the enrolled patients were virtually free of clinically evident acute infection, IL-6 concentrations were widely distributed. An acidic milieu appears to stimulate the production of proinflammatory cytokines and chemokines, providing additional mechanisms leading to more kidney injury, muscle destruction, and wasting. It may be essential to clarify the mechanism behind this. As a measure of inflammation, IL-6 was lower in the intervention group than in the control group after three months; this may be the effect of the correction of acidosis [19]. In this study, highly sensitive CRP values were unavailable for all patients, so we did not include hs-CRP in the analysis.

In the intervention group, predialysis blood pressure and interdialytic weight gain did not show any significance in the observed changes, reflecting no volume retention, despite a minimal increase in plasma sodium level at two periods of the study groups. It was consistent with Movilli et al.'s result that correction of MA by oral bicarbonate supplementation in the range of 1 to 4 g/day does not lead to greater IWG and fluid overload [31]. In this study, a single fixed dose of 500 mg/day was used, and such a low dose may explain the minimum volume retention and blood pressure.

An interesting finding was the reduction in serum potassium in the intervention group compared with the control group, and this was concordant with reports by Melamed and Raphael [20, 32].

Treating acidosis might improve muscle strength and function by reducing muscle breakdown. It improves lower extremity muscle mass and strength and positively affects the physical function and exercise capacity of ESKD/HD. Muscle strength was measured by a Handgrip dynamometer, which increased significantly in the intervention group compared with the control [33]. Furthermore, correction of acidosis did not improve TSF thickness at three months compared to the control [34].

In this study, more frequent arterial blood gas analyses would add a better assessment of the acid-base status of the study groups but laboratory logistics limited this. Accordingly, it has limited dose titration, and only a fixed-dose protocol is used. The dialysis blood flow rates were remarkably low, likely explaining the magnitude of predialysis acidemia, which is a limiting factor that could reflect the local practice of dialysis delivery. In addition, the dietary behavior and protein intake may vary during the 3-month study period, which is another limiting factor. Furthermore, the study design was limited by being not blinded and with no placebo control. Extensive and long-term studies should be conducted to assess the effect of correction of acidosis on other electrolytes and micronutrients, including vitamins and minerals and assess nutritional status and quality of life in CKD/ESKD patients.

In conclusion, even a small dose of oral sodium bicarbonate supplementation improved protein metabolism with no significant interdialytic weight gain nor clinically significant hypokalemia. Furthermore, correcting metabolic acidosis increases serum albumin in HD patients with minimal inflammation.

## Figures and Tables

**Table 1:** Baseline characteristics of the study groups.

	Intervention group	Control group	*p* value
Number	25	24	
Age (years)	47.12 + 18.04	50.46 + 14.24	0.14
Gender (M/F)	10/15 (40%/60%)	12/12 (50%/50%)	0.33
Residence (urban/rural)	19/6 (76%/24%)	19/5 (79.2%/20.8%)	0.6
*ESRD*			
Diabetes mellitus	9/36%	12/50%	
Hypertension	2/8%	4/16.66%	
Chronic glomerular disease	1/4%	2/8.33%	
Cystic disease	3/12%	2/8.33%	
Obstructive uropathy	1/4%	1/4.16%	
Others	9/36%	3/12.5%	
Dialysis vintage (years)	2.32 + 0.627	1.96 + 0.55	0.2
BMI	21.94 + 4.29	22.89 + 5.58	0.5
Weight (kg)	60.28 + 13.7	65 + 15.8	0.26
Systolic Bp (mm Hg)	133.72 + 23.4	149.58 + 31.5	0.05^∗^
Diastolic Bp (mmHg)	81.3 + 12.5	85.29 + 15.4	0.32
Interdialytic weight gain	2.376 + 0.68	1.91 + 1	0.75
Arterial pH	7.26 ± 0.06	7.29 + 0.04	0.27
Serum bicarbonate (mmol/l)	17.57 ± 3.34	18.68 ± 3.29	0.98
Serum potassium (mEq/l)	5.64 ± 0.61	5.19 ± 0.72	0.45
Calcium (mg/dL)	8.25 + 2.2	8.18 + 1.4	0.19
Phosphate (mg/dL)	6.39 + 1.4	6.23 + 1.3	0.4
Alkaline phosphatase (U/L)	366 + 66	242 + 43	0.06
Vitamin D (ng/ml)	15.6 + 8.9	14.4 + 8.1	0.77
iPTH (pg/l)	654 + 115	430 + 58	0.02^∗^
Kt/V	1.26 ± 0.33	1.23 ± 0.27	0.36
S. albumin (g/l)	4.11 ± 0.45	3.95 + 0.55	0.36
nPCR (g/kg per day)	0.90 ± 0.19	0.92 + 0.1	0.91
IL-6 (pg/ml)	16.22 ± 24.11	27.62 ± 47.51	0.179
Handgrip	38.8 ± 18.95	35.84 ± 15.39	0.25
Skinfold thickness (mm)	8.98 ± 5.53	10.92 ± 7.99	0.87

**Table 2:** Clinical, laboratory, and nutritional data of the intervention and the control groups at three months of oral sodium bicarbonate therapy.

	Intervention	Control	*p* value	95% CI	
Weight (kg)	60.70 ± 13.88	65.19 ± 15.71	0.294	−13	4.02
Systolic Bp (mm Hg)	130.28 ± 22.57	145.63 ± 23.93	0.25	−28.7	−1.98
Diastolic Bp (mmHg)	78.64 ± 10.67	85.21 ± 15.00	0.083	−14	0.88
Interdialytic weight gain	2.42 ± 0.64	2.20 ± 1.14	0.405	−0.30	0.75
Arterial pH	7.34 ± 0.43	7.29 ± 0.07	0.008^*∗*^	0.11	0.75
S. bicarbonate (mmol/l)	20.69 ± 2.54	18.9 ± 3.85	0.047^*∗*^	0.17	3.5
S. sodium (mEq/l)	138.00 ± 3.11	133.59 ± 20.34	0.289	−3.86	12.6
S. potassium (mEq/l)	5.00 ± 0.43	5.33 ± 0.63	0.039^*∗*^	−0.63	−0.01
Calcium (mg/dL)	8.08 + 2.04	7.79 + 1.55	0.42	−0.74	1.32
Phosphate (mg/dL)	5.56 + 1.28	6.44 + 1.05	0.23	−1.56	−0.2
Alkaline phosphatase (U/L)	312 + 243	308 + 232	0.83	−132	140
Vitamin D (ng/ml)	21.72 + 5.9	11.89 + 6.1	0.06	0.9	18.7
iPTH (pg/l)	437 + 353	551 + 350	0.79	−316	88.4
S. albumin (g/l)	4.15 ± 0.49	3.79 ± 0.47	0.011^*∗*^	0.08	0.63
nPCR (g/kg/day)	1.06 + 0.27	0.90 + 0.27	0.033^*∗*^	−0.26	−0.01
IL-6 (pg/ml)	12.34 ± 10.62	22.88 ± 16.79	0.043^*∗*^	−18.68	−2.37
Kt/V	1.25 + 0.3	1.24 + 0.22	0.6	0.15	0.4
Handgrip	45.01 ± 19.19	33.93 ± 15.06	0.030^*∗*^	1.13	21.02
Skinfold thickness (mm)	10.26 ± 4.89	10.50 ± 7.97	0.899	−4.02	3.54

**Table 3:** Repeated measures ANOVA for outcome variables.

Outcome variables^†^	*p* value
Serum albumin	0.19
nPCR	0.29
IL-6	0.29
SFT	0.01^*∗*^
Handgrip	0.06

^†^For all variables, the sphericity assumption was not violated.

**Table 4:** Primary endpoints.

	Intervention, no (%)	Control, no (%)	RR (95% CI)	*p* value
Serum albumin (mg/dl) at 3 m	15/25 (60%)	4/24 (16.6%)	3.75 (1.4 to 9.7)	0.006
nPCR (g/kg/day) at 3 m	14/25 (56%)	3/24 (12.5%)	4.84 (1.47 to 13.64)	0.008

## Data Availability

All data will be available on e-mail request, as we should have institutional approval first.
